# Supplementary material for: The Functional Characteristics and Soluble Expression of Saffron CsCCD2
Source: Int J Mol Sci. 2023 Oct 11;24(20):15090. doi: 10.3390/ijms242015090 (PMC10606151; doi:10.3390/ijms242015090)
Supplement: Supplementary file 1 [file ijms-24-15090-s001.zip › Supplementary data-revised.pptx]

## Slide 1
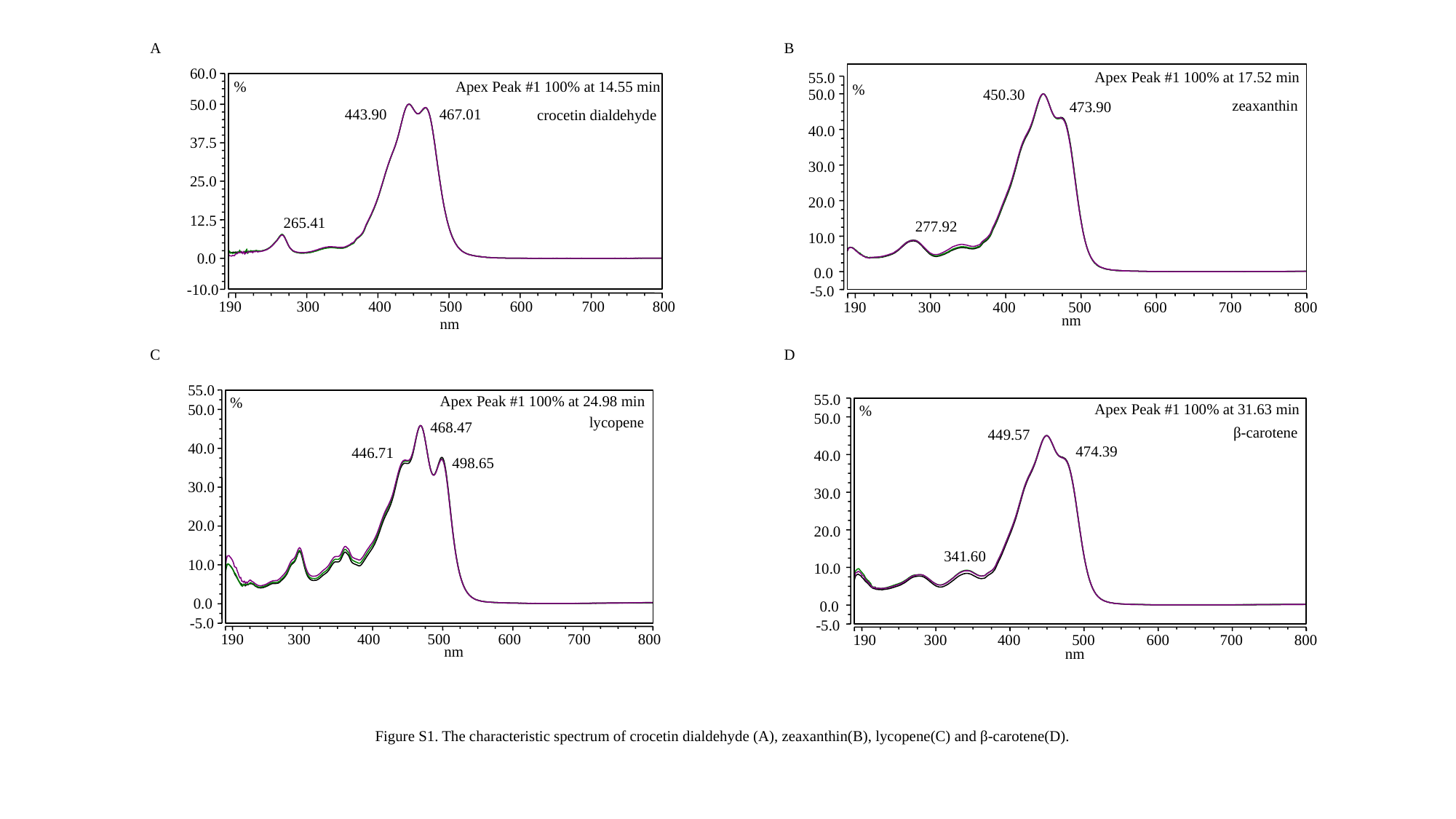

B
A
60.0
Apex Peak #1 100% at 17.52 min
55.0
%
Apex Peak #1 100% at 14.55 min
%
450.30
50.0
zeaxanthin
50.0
473.90
crocetin dialdehyde
467.01
443.90
40.0
37.5
30.0
25.0
20.0
12.5
265.41
277.92
10.0
0.0
0.0
-10.0
-5.0
190
300
400
500
600
700
800
190
300
400
500
600
700
800
nm
nm
C
D
55.0
55.0
Apex Peak #1 100% at 24.98 min
%
Apex Peak #1 100% at 31.63 min
50.0
%
lycopene
50.0
468.47
β-carotene
449.57
40.0
474.39
446.71
40.0
498.65
30.0
30.0
20.0
20.0
341.60
10.0
10.0
0.0
0.0
-5.0
-5.0
190
300
400
500
600
700
800
190
300
400
500
600
700
800
nm
nm
Figure S1. The characteristic spectrum of crocetin dialdehyde (A), zeaxanthin(B), lycopene(C) and β-carotene(D).

## Slide 2
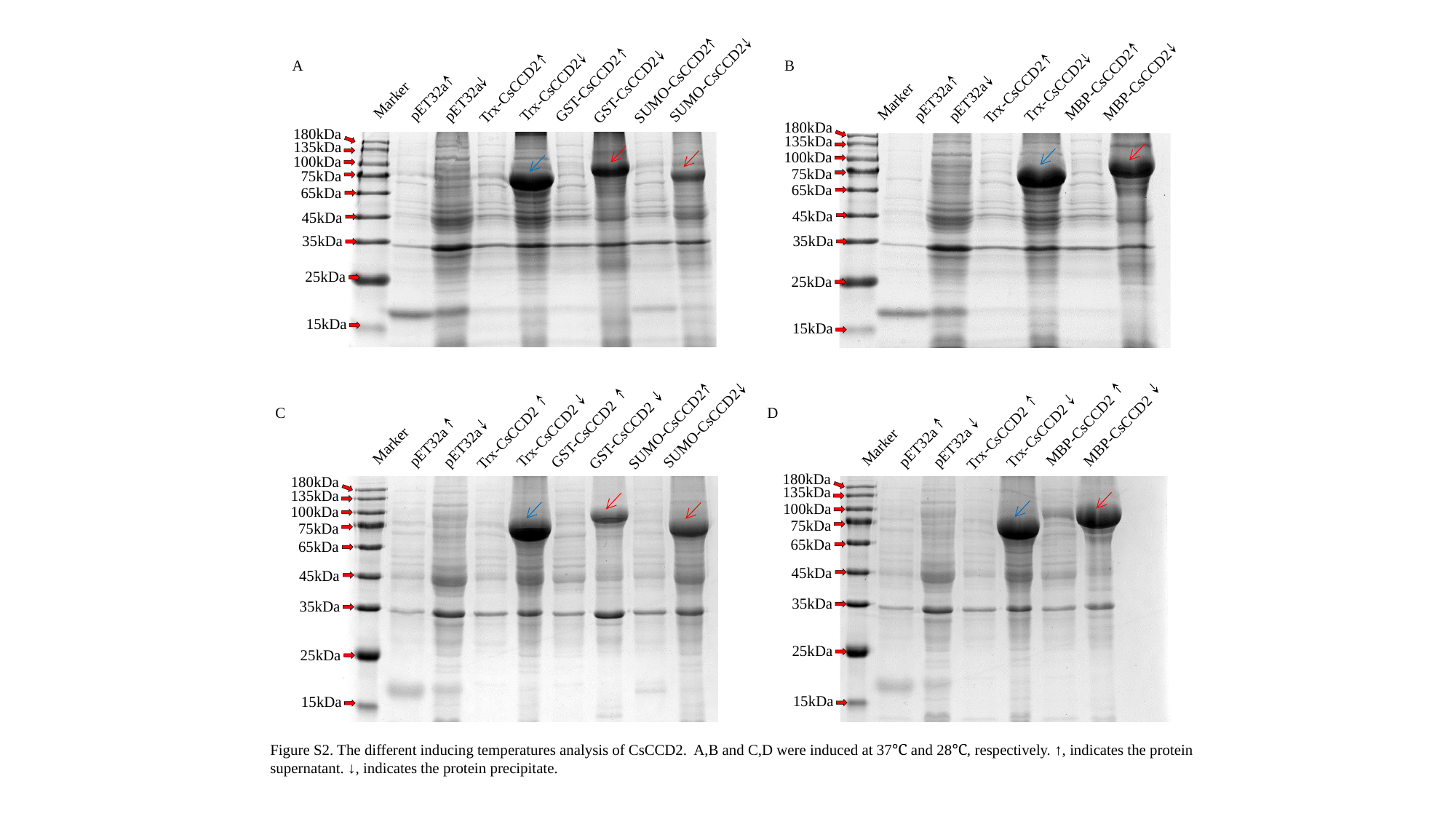

A
B
MBP-CsCCD2
SUMO-CsCCD2
GST-CsCCD2
MBP-CsCCD2
SUMO-CsCCD2
Marker
Marker
GST-CsCCD2
Trx-CsCCD2
Trx-CsCCD2
Trx-CsCCD2
Trx-CsCCD2
pET32a
pET32a
pET32a
pET32a
180kDa
135kDa
180kDa
135kDa
100kDa
100kDa
 75kDa
 75kDa
 65kDa
 65kDa
 45kDa
 45kDa
 35kDa
 35kDa
25kDa
25kDa
 15kDa
 15kDa
MBP-CsCCD2
MBP-CsCCD2
Marker
Trx-CsCCD2
Trx-CsCCD2
pET32a
pET32a
C
D
SUMO-CsCCD2
GST-CsCCD2
SUMO-CsCCD2
Marker
GST-CsCCD2
Trx-CsCCD2
Trx-CsCCD2
pET32a
pET32a
180kDa
135kDa
180kDa
135kDa
100kDa
 75kDa
 65kDa
 45kDa
 35kDa
100kDa
 75kDa
 65kDa
 45kDa
 35kDa
25kDa
25kDa
 15kDa
 15kDa
Figure S2. The different inducing temperatures analysis of CsCCD2. A,B and C,D were induced at 37℃ and 28℃, respectively. ↑, indicates the protein supernatant. ↓, indicates the protein precipitate.

## Slide 3
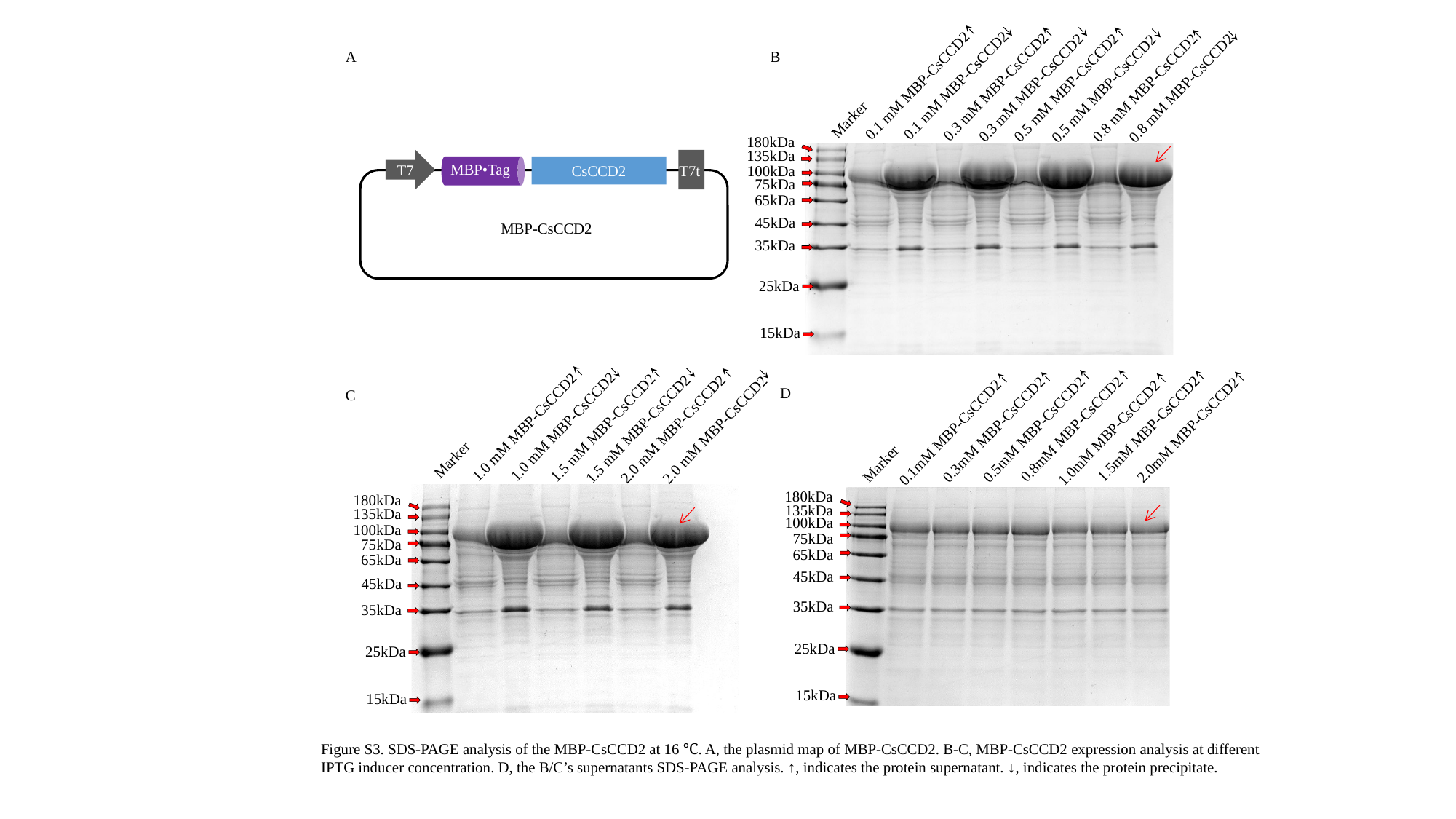

A
B
0.1 mM MBP-CsCCD2
0.3 mM MBP-CsCCD2
0.8 mM MBP-CsCCD2
0.1 mM MBP-CsCCD2
0.5 mM MBP-CsCCD2
0.3 mM MBP-CsCCD2
0.8 mM MBP-CsCCD2
0.5 mM MBP-CsCCD2
Marker
180kDa
135kDa
T7
MBP•Tag
T7t
CsCCD2
MBP-CsCCD2
100kDa
 75kDa
 65kDa
 45kDa
 35kDa
25kDa
 15kDa
D
C
1.5mM MBP-CsCCD2
0.8mM MBP-CsCCD2
0.5mM MBP-CsCCD2
0.3mM MBP-CsCCD2
1.0mM MBP-CsCCD2
0.1mM MBP-CsCCD2
2.0mM MBP-CsCCD2
1.0 mM MBP-CsCCD2
1.5 mM MBP-CsCCD2
1.0 mM MBP-CsCCD2
1.5 mM MBP-CsCCD2
2.0 mM MBP-CsCCD2
2.0 mM MBP-CsCCD2
Marker
Marker
180kDa
135kDa
180kDa
135kDa
100kDa
100kDa
 75kDa
 75kDa
 65kDa
 65kDa
 45kDa
 45kDa
 35kDa
 35kDa
25kDa
25kDa
 15kDa
 15kDa
Figure S3. SDS-PAGE analysis of the MBP-CsCCD2 at 16 ℃. A, the plasmid map of MBP-CsCCD2. B-C, MBP-CsCCD2 expression analysis at different IPTG inducer concentration. D, the B/C’s supernatants SDS-PAGE analysis. ↑, indicates the protein supernatant. ↓, indicates the protein precipitate.
